# Precision, Applicability, and Economic Implications: A Comparison of Alternative Biodiversity Offset Indexes

**DOI:** 10.1007/s00267-021-01488-5

**Published:** 2021-06-07

**Authors:** Johanna Kangas, Peter Kullberg, Minna Pekkonen, Janne S. Kotiaho, Markku Ollikainen

**Affiliations:** 1 Department of Economics and Management, University of Helsinki, Helsinki, Finland; 2 Finnish Environment Institute, Helsinki, Finland; 3 Department of Biological and Environmental Sciences, University of Jyväskylä, Jyväskylä, Finland; 4 School of Resource Wisdom, University of Jyväskylä, Jyväskylä, Finland

**Keywords:** Biodiversity offsetting, Ecological compensation, No net loss, Trade ratio, Biodiversity calculation method

## Abstract

The rates of ecosystem degradation and biodiversity loss are alarming and current conservation efforts are not sufficient to stop them. The need for new tools is urgent. One approach is biodiversity offsetting: a developer causing habitat degradation provides an improvement in biodiversity so that the lost ecological value is compensated for. Accurate and ecologically meaningful measurement of losses and estimation of gains are essential in reaching the no net loss goal or any other desired outcome of biodiversity offsetting. The chosen calculation method strongly influences biodiversity outcomes. We compare a multiplicative method, which is based on a habitat condition index developed for measuring the state of ecosystems in Finland to two alternative approaches for building a calculation method: an additive function and a simpler matrix tool. We examine the different logic of each method by comparing the resulting trade ratios and examine the costs of offsetting for developers, which allows us to compare the cost-effectiveness of different types of offsets. The results show that the outcomes of the calculation methods differ in many aspects. The matrix approach is not able to consider small changes in the ecological state. The additive method gives always higher biodiversity values compared to the multiplicative method. The multiplicative method tends to require larger trade ratios than the additive method when trade ratios are larger than one. Using scoring intervals instead of using continuous components may increase the difference between the methods. In addition, the calculation methods have differences in dealing with the issue of substitutability.

## Introduction

Biodiversity offsetting is a conservation tool designed to compensate for the loss of biodiversity caused by development projects (McKenney and Kiesecker 2010). It requires that whenever developers degrade biodiversity, comparable ecological gains must be provided elsewhere so that the lost ecological value is compensated for. The principle of no net loss of biodiversity (NNL) is commonly associated with biodiversity offsets (OECD 2016). Offsets are designed to compensate for unavoidable biodiversity loss only, which means that in accordance with a mitigation hierarchy, developers must always avoid and minimize harm and restore biodiversity on the development site first (McKenney and Kiesecker 2010; see Moilanen and Kotiaho 2020 for a suggestion of a modified mitigation hierarchy). Currently, offsetting schemes are becoming increasingly popular as a way to complement the policy mix for biodiversity conservation (Bull and Strange 2018; zu Ermgassen et al. 2019b). These initiatives face, however, multiple sources of uncertainty and ecological risks as well as several theoretical and practical challenges (e.g., Bull et al. 2013; Maron et al. 2016). Among other things, difficulties in measuring biodiversity and matching ecological losses and gains, are one of the most problematized issues in scientific biodiversity offsetting literature (Gardner et al. 2013; Gonçalves et al. 2015; Guillet and Semal 2018; Quétier and Lavorel 2011; zu Ermgassen et al. 2019a).

To meet the objective of NNL, both ecological losses from development and gains from compensation projects need to be estimated and balanced. Calculation methods are used to describe how much compensation in each case is needed to achieve NNL. A trading ratio determines the offset requirement: it calculates the area of compensation site(s) required by matching the ecological value of loss and gain. The trade ratio can be adjusted with multipliers to increase the offset requirement to account for factors such as risks and uncertainties regarding the success of restoration, time lags, leakage, flexibility, spatial issues, and other important aspects of offsetting (Laitila et al. 2014; Moilanen and Kotiaho 2018; Moilanen et al. 2009). However, some of these issues could be better addressed with complementary approaches instead of multipliers (e.g., temporal issues can be overcome with habitat banking) (Bull et al. 2017).

A large number of indicators have been developed to measure different aspects of biodiversity (e.g., Juutinen and Mönkkönen 2004; Morris et al. 2014). In biodiversity offsetting, the indicators and the calculation method must reflect what the offsetting mechanism is targeted at and balance trade-offs between feasibility and ecological robustness. The choice has an effect on the outcomes for biodiversity as different metrics and methods lead to different offsetting requirements (Bull et al. 2014a; Gonçalves et al. 2015; Quétier and Lavorel 2011). In many cases, offsets have been measured based on area alone, i.e., a given degraded area is compensated with a similar-sized restored or preserved area of the same habitat type (Quétier and Lavorel 2011). Crude methods are usually simpler and less costly to implement, but as such methods ignore variations in an ecosystem quality, there is always a risk that some biodiversity features are overlooked and lost (OECD 2016).

Compound methods, which supplement area-based measurements with ecological information on different biodiversity features, are considered to be preferable (Bull et al. 2013). Different attributes of biodiversity can be combined to measure the condition of a given type of ecosystem. Well-designed attributes can ideally capture all components of biodiversity that the offsetting scheme aims to protect. There is a trade-off between the benefits of including more precision in the calculation method and the efficiency and cost-saving gains of simpler methods (Lave et al. 2010; Needham et al. 2019). This trade-off affects the choice of components included in the method (Gamarra et al. 2018).

There are few scientific articles that numerically compare different calculation methods and metrics in the context of biodiversity offsetting. Bull et al. (2014a) provide numerical calculations with different existing methods: they compared the approaches by calculating the required offset gains with a case study from gas extraction projects in Uzbekistan and found that the methods result in various different requirements for the gain to offset the same development. More recently, Marshall et al. (2020) compared the performance of four metrics by measuring the development impacts and offset requirements, simulating the development and subsequent offsetting, and linking the simulations to population viability models for three species. They found that benefits delivered by offsets are species-specific, targeting offsets to habitats does not necessarily lead to species persistence, and species benefit more likely when impacts are avoided rather than offset. However, several papers have identified and analyzed existing methods qualitatively in order to recognize the needs for further development (Bezombes et al. 2017; Marshall et al. 2019; Quétier and Lavorel 2011) and aimed to help offsetting practitioners to choose the right calculation method (Gamarra et al. 2018; Knight et al. 2019).

We add to the literature by comparing numerically three different approaches to develop an offset calculation method: a multiplicative or additive function that combines multiple indicators or a simpler matrix method. A method called ELITE index identifies biodiversity components important for different ecosystems, assumes multiplicative effects of the components, and uses them to calculate an ecological index value (Kotiaho et al. 2015, 2016; Mustajärvi et al. 2019). By using the same biodiversity components, we build an additive calculation method and a matrix method and compare the results. We use Habitat Hectares (HH) developed in Australia as an example for the additive approach and an offset scoring matrix (updated version called Biodiversity metric 2.0) developed originally for offsetting pilots in the UK as an example for the matrix method. The comparison between the calculation methods is performed using ecosystem data from Southern Finland. We assume a hypothetical offsetting case and calculate both ecological losses at a development site and gains at a compensation site with each of the three methods. We compare the trade ratios and relate those to the area needed to offset the losses. We complement this comparison by examining how the trade ratios derived from the different calculation methods lead to differences in costs of offsetting when there are different options to produce the offset. Thus, we can compare the cost-effectiveness of the passive measure of conservation and active restoration measures.

## Calculation Methods for Measuring Biodiversity in the Context of Biodiversity Offsetting

Here we extend the ELITE index and use the components of the index for building the additive and matrix methods for Finnish ecosystems based on the HH and the UK approaches.

### A Multiplicative Calculation Method

The ELITE index was originally developed for estimating the degree of degradation compared to the natural state of ecosystems in Finland (Kotiaho et al. 2016). The state of an ecosystem is calculated using a few ecosystems type-specific, ecologically most relevant structural components. These key components important for biodiversity were identified based on expert assessments. The latest scientific understanding as well as the availability of data affected the choice of the structural components. For each structural component, the current state is compared to a pre-defined reference state. The reference state represents the pre-degradation state of the ecosystem in question, also known as the natural state.

Weights are attributed to each component in the ELITE index. They represent the fraction of the ecosystem’s overall condition lost if the component in question is completely degraded. The weights are set because different ecological components have different overall influences on the ecological state of the ecosystem and in most cases, the site’s condition is not zero even if there is a complete degradation of one of its components (Kotiaho et al. 2016). Similarly, restoration of one component does not usually lead to the complete recovery of the ecosystem. The index value for the overall state ranges from 0 to 1, where 1 is the ecosystem condition in the natural state (reference state) and 0 implies that an ecosystem is completely degraded.

1 $$R^E = \frac{{\mathop {\prod }\nolimits_{n = 1}^{N^E} \left( {1 - L_n^E\left( {1 - \frac{{n_{curr}}}{{n_{ref}}}} \right)} \right) - \mathop {\prod}\nolimits_{n = 1}^{N^E} {\left( {1 - L_n^E} \right)} }}{{1 - \mathop {\prod }\nolimits_{n = 1}^{N^E} \left( {1 - L_n^E} \right)}}$$

Equation (1) calculates the condition of a given ecosystem, *R*^*E*^, by assuming multiplicative effects of the components. *N*^*E*^ denotes the number of structural components in the ecosystem *E* and $L_n^E$ represents the weight of each component *n* in the ecosystem *E*. *n*_*curr*_ and *n*_*ref*_ denote the current and the reference state of component *n* respectively. The lower bound of the original index proposed by Kotiaho et al. (2015) is dependent on the weights and is always larger than zero. For comparison purposes, here we have rescaled the index to fall between 0 and 1. $\mathop {\prod}\nolimits_{n = 1}^{N^E} {( {1 - L_n^E( {1 - \frac{{n_{curr}}}{{n_{ref}}}} )} )}$ is the original index formulation. $\mathop {\prod}\nolimits_{n = 1}^{N^E} {\left( {1 - L_n^E} \right)}$ is the minimum value of the original index, and thus the whole formula scales the original index to range from 0 to 1 when the difference is divided by term $1 - \mathop {\prod}\nolimits_{n = 1}^{N^E} {\left( {1 - L_n^E} \right)}$.

We extend the index from Kotiaho et al. (2015, 2016) by dividing originally one component, the amount of decaying wood, into three components. The amount of decaying wood is one of the most important factors affecting the diversity of forest species (Kotiaho et al. 2016): in natural forests, there is plenty of deadwood in all stages of succession from different tree species, diameters and degrees of decay, as well as continuity in the availability of dead wood, and altogether more than 4000 species in Finland are directly or indirectly dependent on dead wood (Siitonen 2001). For this reason, the amount of deadwood in the original version and the different decay stages together here are given the largest weight in the index.

With the division to finer categories, we aim to increase the accuracy of the index. As we have divided one component into three, the relative sizes of the weights have been updated and new reference values have been set. All weights have been scaled in order to keep their interpretation similar to the original method after the modification of Eq. (1).

Components for large trees (with a diameter of at least 40 cm) and broad-leaved trees are set to reflect the importance of the structural diversity of the tree stand. Large trees are significant especially for predator birds and epiphytes, and they produce important large-sized decaying wood (Kotiaho et al. 2016). Broad-leaved trees, such as birches, goat willow, and especially aspen, increase the species diversity of forests significantly (Kouki et al. 2004; Tikkanen et al. 2006). Table 1 represents the original and new versions of the ELITE index for sub-xeric, mesic, and herb-rich heath forests, which belong to the forest ecosystems in the ELITE index and are thus assessed by using the same components, reference values, and weights. The ecosystems in our numerical analysis belong to this type of forest.

**Table 1:** The original and extended ELITE index components, their weights, and reference values for sub-xeric, mesic, and herb-rich heath forests (source for the original reference values and weights: Kotiaho et al. 2015)

Structural components	Weights		Reference values	
	Original	Extended	Original	Extended
Decaying wood (m^3^/ha)	0.6		80	
Decaying wood, stage 1 (m^3^/ha)		0.17		26
Decaying wood, stage 2 (m^3^/ha)		0.17		26
Decaying wood, stage 3 (m^3^/ha)		0.17		26
Broad-leaved trees (m^3^/ha)	0.4	0.28	50	50
Large trees (≥40 cm, pcs/ha)	0.4	0.28	20	20

### An Additive Calculation Method

As the second biodiversity offset calculation method, we consider the possibility to use an additive function instead of a multiplicative one. An example of this kind of method is the Australian HH, which was originally developed for offsetting native vegetation areas in Victoria (DSE 2004; Parkes et al. 2003). Nowadays, several different methods are based on the HH approach (OECD 2016). Similar to the ELITE index, HH is a compound method that assesses vegetation quality against a reference state in a given vegetation type. The maximum score of each component has a similar role to weights in the ELITE index. We compare the additive method to the multiplicative one by using the components and deriving the maximum scores from the weights of the ELITE index. As HH does not use raw input data but instead, converts the values of components into scores based on scoring intervals, we convert the raw data used in ELITE into scores by using a similar logic for the scoring as in HH (DSE 2004). The value is calculated as an arithmetic sum through pre-defined components and their scores:

2 $$R^H = \mathop {\sum}\limits_{i = 1}^h {\alpha _i}$$

where the condition of a given ecosystem, *R*^*H*^, is calculated by assuming additive effects of the components. Parameter *h* is the number of structural components in the ecosystem *H*. Parameter *α*_*i*_ is the score of a given component and is determined based on scoring intervals, which are explained in more detail in Online Resource 1.

### A Matrix Method

As a third calculation method, we examine a simpler matrix approach. This kind of method was developed for an offsetting pilot in the UK (DEFRA 2012). The method quantifies the value of habitats as a product of habitat distinctiveness and condition scores:

3 $$B_{ha} = D_{hab} \cdot C_{hab}$$

where *B*_*ha*_ is the number of biodiversity units per hectare, *D*_*hab*_ is the distinctiveness score of the habitat (6, 4, or 2 for high, medium, and low) and *C*_*hab*_ is habitat condition score (3, 2, or 1 for good, moderate or poor) (McVittie and Faccioli 2020).

We convert the ELITE index into a condition score. The distinctiveness categories are adapted to the Finnish ecosystems based on a report examining the suitability of Finnish habitats for biodiversity offsetting (Raunio et al. 2019). The sites used in the analysis are assigned to the same distinctiveness category. Thus, this approach simply scores the result of the multiplicative method into three categories. To make the results comparable, we scale this method between 0 and 1, the same as the multiplicative and additive methods. Categories and scoring of the habitat distinctiveness and condition can be found in Online Resource 1.

### General Properties of the Multiplicative and Additive Methods

In this section, we explore the general properties of the multiplicative and additive methods. We show that the additive method gives always higher biodiversity values compared to the multiplicative one. We further show that the multiplicative method tends to require larger trade ratios when the average loss at the development site is higher than the average gain at the compensation site. Here we focus on the aggregation method itself and assume that both methods use the same continuous component values as inputs. Thus, the additive method does not use scoring intervals as in the subsequent analysis. Under this assumption, the additive method is simply a weighted arithmetic mean of the input values as given by Eq. (4).

4 $$R^H = \mathop {\sum}\limits_{n = 1}^N {\frac{{L_nx_n}}{{\mathop {\sum}\limits_{n = 1}^{N} {L_n} }}}$$

In Eq. (4), the condition of a given ecosystem is *R*^*H*^*, N* denotes the number of components, *L*_*n*_ represents the weight of component *n*, and *x*_*n*_ denotes the input value of component *n*.

#### Proof

To simplify the equations, we denote a vector **X** of *N* indicator values $\frac{{n_{curr}}}{{n_{ref}}}$ with ${\mathbf{X}}\,\left\{ {x_i \in {\Bbb R}\left| {0 \le {\mathrm{x}} \le 1} \right.} \right\}$. For simplicity, we further assume that all component weights *L* are exactly 1. This simplifies the multiplicative method (Eq. (1)) to a simple product of the input values **X** given by Eq. (5).

5 $$R^E = \mathop {\prod}\limits_{n = 1}^N {\left( {x_n} \right)}$$

1. For all **X**, $R^E\left( {\boldsymbol{X}} \right) \le {\mathrm{min}}\left( {\boldsymbol{X}} \right)$. To explain this, consider the smallest value in **X**. When it is multiplied with the other values of **X**, the product will always be smaller than the minimum itself, because the multipliers are, by definition, always smaller than one.

2. For all X, $R^H\left( {\boldsymbol{X}} \right) \ge {\mathrm{min}}\left( {\boldsymbol{X}} \right)$. The arithmetic mean of the minimum of **X** and all other values **X** have to be larger than the minimum because the other values are by definition larger than min(***X***).

3. By transitivity *R*^*E*^(***X***) ≤ *R*^*H*^(***X***).

#### Numerical illustration

**Fig. 1:**
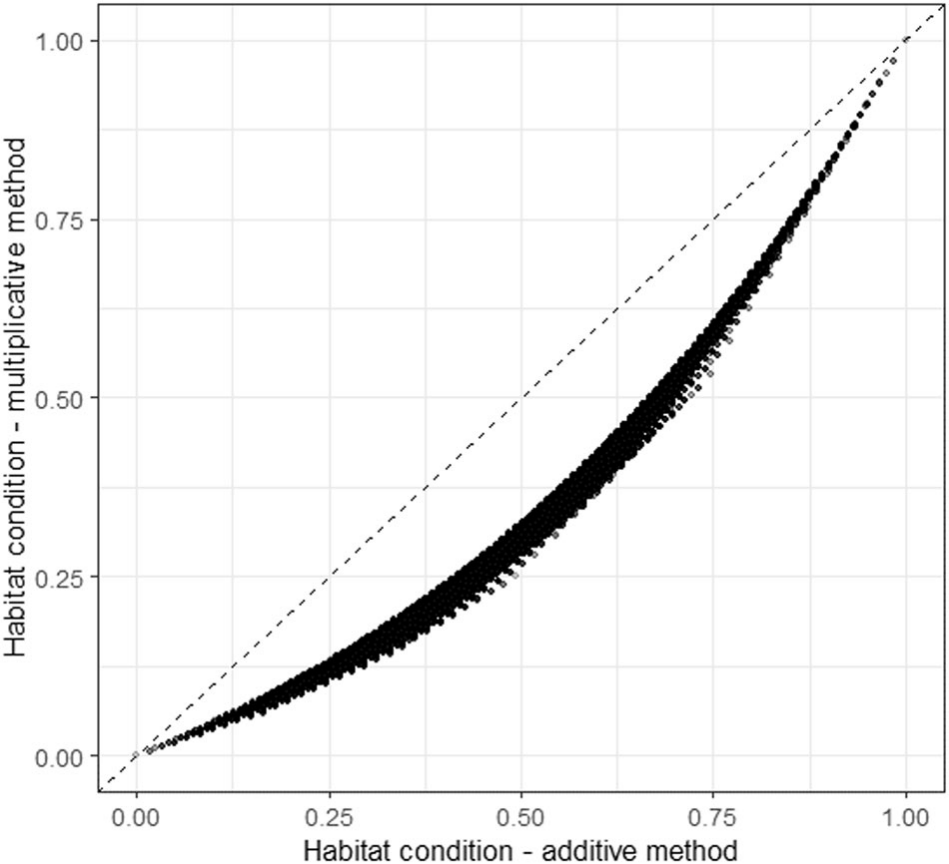
Numerical comparison of the methods, multiplicative method on the *y*-axis and additive one on the *x*-axis

Here we explore the two methods numerically. We created a matrix containing all possible combinations of the five components when they range from 0 to 1, with increments of 0.1. This created all together 161,051 unique value combinations ranging from the situation where all components are zero, to a situation where all features have reached the reference condition and have value 1. We computed the overall state for all possible combinations of the component input values using both the multiplicative and additive methods.^1^ Fig. 1 compares the methods. The diagonal dashed line illustrates a situation where both methods would provide the same outcomes. Each black dot represents one value combination. All dots are below the dashed line indicating that with similar variable combinations the multiplicative method gives always lower biodiversity values compared to the additive one.

#### Implications for biodiversity offsetting

**Fig. 2:**
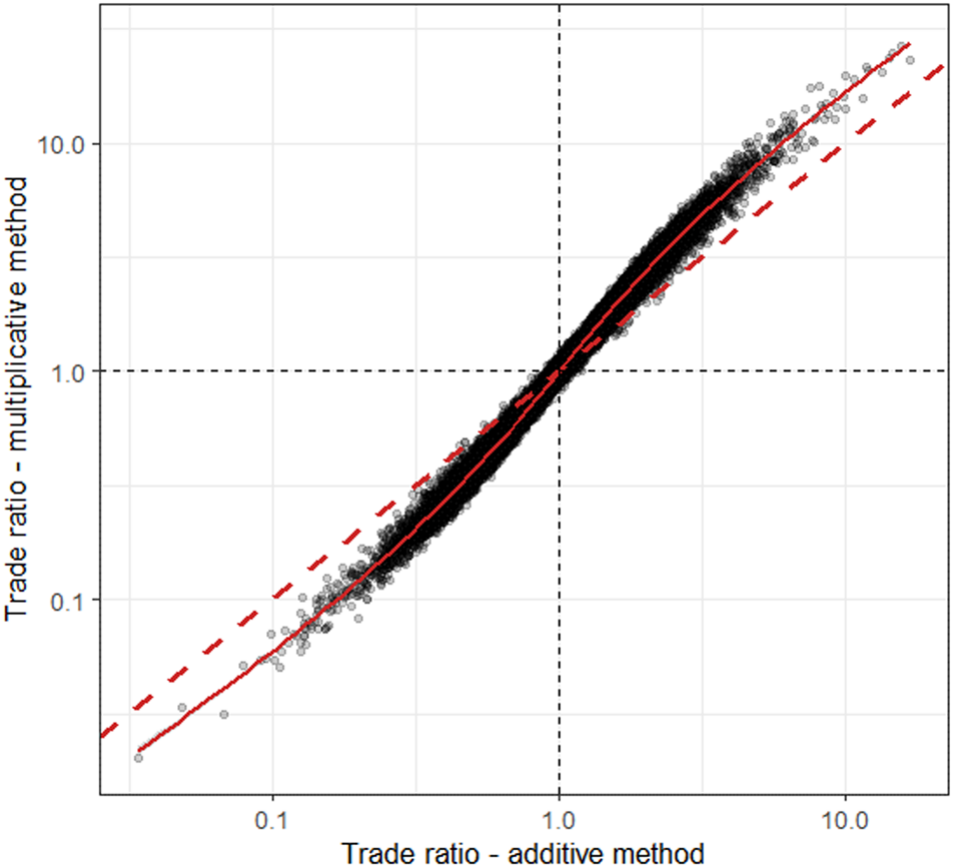
Trade ratios computed for 50,000 randomly selected development and compensation sites, multiplicative method on the *y*-axis and additive one on the *x*-axis on a logarithmic scale

The curved relationship of the two methods also influences the offset requirements in different situations. Figure 2 shows trade ratios computed for 50,000 randomly selected development and compensation sites that are drawn from the same value combinations that were used in Fig. 1. The dashed red line indicates where the trade ratios are similar regardless of the computation method. If the dot is above the line, the multiplicative method requires higher trade ratios, and if the dot is below the line, the additive method requires higher trade ratios. The solid red line is a smoothed average of the realized trade ratios based on the random draw of sites. Figure 2 shows that the offset requirements differ depending on whether the trade ratios are below or above 1. When the average loss per unit area at the development site is higher than the average gain produced per unit area at the compensation site (trade ratio is larger than one), in a majority of the cases, the multiplicative method is expected to require higher trade ratios than the additive method. If the offset gain is higher than the loss at a development site, the situation is reversed. Thus, the shape of the solid red line is convex below 1 and concave above 1.

## Data and Methods

### Hypothetical Offsetting Case and Scenarios

We analyze the calculation methods by comparing offset trade ratios in a hypothetical offsetting case. The data is from two sites situated in Southern Finland, one of which is considered as the hypothetical development site and the other as the hypothetical compensation site. The data is collected by Parks and Wildlife Finland, which is a part of the state-owned organization Metsähallitus and manages state-owned nature reserves in Finland (SAKTI 2018). The sites belong to the same group of ecosystems in the ELITE index and are thus assessed by using the same components, reference values, and weights.

**Fig. 3:**
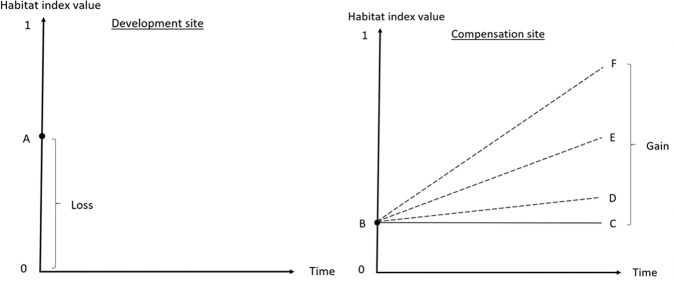
The procedure of calculating the amount of loss due to development and the gain from producing offsets: the current states of the hypothetical development site (**A**) and the compensation site (**B**) are calculated with the data, and the amount of gain is the difference between the baseline (**C**) and three compensation scenarios (**D**, **E**, **F**)

The procedure of the calculation is illustrated in Fig. 3, where the development site is represented in the panel on the left and the compensation site on the right. Linear curves are used just for illustration. The current states of both the development and compensation sites (A, B) are calculated using each method. Data for the components used in the calculation methods are represented in Table 2. We postulate an exogenous loss and assume that when the site is developed, its condition drops to zero. Thus, the loss equals the current state of the development site. Also, we assume that the area of the development site is 1 ha. The time period of the calculations is 30 years.

**Table 2:** Data for the structural components from the development and compensation sites (current states) and three compensation scenarios over the 30-year time period

	Development site	Compensation site			
	Current state	Current state	Conservation	Dead wood creation	Burning
Dead wood, stage 1	14	3	7	7	26
Dead wood, stage 2	11	3	6	26	26
Dead wood, stage 3	11	1	8	26	26
Broad-leaved trees	49	9	9	14	24
Large trees	18	10	10	10	10

To get a better insight into the implications of choosing a certain calculation method, we have three different scenarios for the compensation site: 1. conservation, 2. conservation and restoration with deadwood creation, or 3. conservation and restoration with prescribed burning (D, E, F in Fig. 3). The gain is calculated as the difference between the compensation scenario and a baseline (C) (e.g., Maron et al. 2013). Although the additive and matrix methods are applied in a policy context where governments and landowners make fixed management contracts, we assume that conservation guarantees a permanent gain with all the methods. Restoring degraded forests by creating dead wood where it is scarce aims to increase the quantity, quality, and continuity of decaying wood closer to the amount typical for natural forests, which strengthens the populations of deadwood-dependent species (Elo et al. 2019; Hekkala et al. 2016). Controlled burning is considered the most effective way to restore heathland forests, but it is costly (Similä and Junninen 2012; Penttilä et al. 2013). The fire increases the amounts of charred and decaying wood, affects the quality of wood in living trees, supports the continuity of decaying wood as damaged trees die gradually even years after the fire, and adds to the diversity of tree structure, for instance, by increasing the volume of broad-leaved trees. (Eales et al. 2018; Kuuluvainen et al. 2002).

The trade ratio determines the amount of compensation area required and is adjusted to match the amount of loss to the gain as follows (e.g., Laitila et al. 2014):

6 $$\sigma = \frac{L}{{\mathrm{G}}},$$

where *σ* is the trade ratio, *L* is the amount of loss and *G* is the amount of gain. Equation (6) matches the ecological value of loss and gain assuming certain offset outcomes. Multipliers are used to adjust the trade ratio to account for time lags and other sources of uncertainty. Each of the calculation methods has a different practice for employing multipliers, dependent on the policy context where they are used (adjusted trade ratios for each method in Online Resource 2). We examine the impact of multipliers on the results briefly in “Offset Requirements with Adjusted Trade Ratios”.

### Estimates for the Scenarios

To calculate the amount of gain in different compensation scenarios, we must assess how the biodiversity features, measured with the components of the methods, develop over the 30-year time period. These assessments are based on literature. To cover all the scenarios, we must assess both the natural succession of the ecosystem (conservation scenario) and the impacts of the restoration measures. As our calculations are based on hypothetical offsetting cases, we use rough estimates in assessing the development of the components. They suffice to facilitate our analysis for comparing the different methods. However, in real-life offsetting cases, expert assessments and possibly detailed forest simulations should be used to assess the future gains of restoration when used as offsets. Also, the trade ratio should be adjusted with appropriate multipliers to account for the uncertainty of the estimations (Moilanen and Kotiaho 2018). In “Sensitivity analysis”, we perform sensitivity analysis to account for the uncertainty of restoration outcomes.

Table 2 shows how the components are estimated to evolve under different scenarios. With only conservation, we assess the natural accumulation of decaying wood based on Tuhus (1997). The amount of dead wood in each decay class as well as the rate of decaying is assessed based on Mäkinen et al. (2006) and Heikkilä et al. (2008). We assume that dead wood stays 15 years in stage 1, 10 years in stage 2, and 17 years in stage 3. With dead wood creation, we assume that 20 m^3^ ha^−1^ of decaying wood is artificially created twice, immediately and after 10 years (Similä and Junninen 2012). With prescribed burning, the amount of dead wood increases substantially as on average 50% of the tree stand dies over time (Similä and Junninen 2012) and thus, the reference values are achieved in each decay class during the 30-year time period.

For broad-leaved and large trees in the conservation scenario, we make a simplifying assumption that their volume and number remain constant. The production of dead wood supports the growth of broad-leaved trees and thus, their volume increases. With prescribed burnings, the increase is higher (Kuuluvainen et al. 2002). The figures for broad-leaved trees are rough estimates, as we know that their volume increases (Similä and Junninen 2012), but the exact figures are difficult to estimate. The number of large trees is kept constant over the 30-year time period since the restoration measures do not have an impact on their number.

To sum up the scenarios and their impact on the components of the methods, with conservation, only the amount of dead wood increases moderately, whereas, with the restoration, we find an increase in all components, apart from large trees, either to some degree (creating decaying wood) or very effectively (prescribed burning). These aspects bring out the different qualities of the calculation method in the next section.

The choice of a baseline (C in Fig. 3) impacts greatly the calculation results for the gain and trade ratios, and consequently, whether the goal of NNL is achieved (Bull et al. 2014b; Gordon et al. 2011). We use a fixed baseline against which the gain is calculated, i.e., we have a simplifying assumption that the state would remain constant in the business-as-usual scenario. A more likely scenario would be that the ecological state decreases due to forestry. However, the baseline that would predict the future trend in the state is inherently more uncertain, and overestimating future biodiversity decrease leads to lower offset requirements and the risk of net loss (Bull et al. 2014b). In the long run, measuring gains against a relative, decreasing baseline leads to a declining trend of biodiversity overall, although at a slower rate than without offsetting, whereas a fixed baseline would halt the declining trend (Simmonds et al. 2020). In “Sensitivity analysis”, we perform sensitivity analysis to examine the implications of different baselines to the offset requirements. The conservation scenario represents a so-called averted-loss offset, which is related to several problems in achieving biodiversity outcomes (Moilanen and Laitila 2016; zu Ermgassen et al. 2019b). In our analysis, we do not take into account these aspects.

### The Costs of Conservation and Restoration

We compare the costs of offsetting for the hypothetical developer in the different scenarios. We calculate the costs, derived from the costs of conservation and restoration, and examine the implications of choosing a given calculation method. In our analysis, the cost of conservation is estimated to be 3000 € ha^−1^, which is calculated as the Faustmannian discounted bare land value for heath forests in Southern Finland, administrative costs of establishing a nature reserve added (Kangas and Ollikainen 2019). The costs of producing dead wood is estimated to be 500 € ha^−1^ (Hyvärinen and Aapala 2009; Kotiaho et al. 2016). Restoration burning is expensive in comparison with other restoration measures: the costs per hectare are ~1500 € ha^−1^ on average (Lindberg et al. 2018).

## Results

### Offset Requirements in Different Scenarios

First, we calculate the current ecological state of the hypothetical development and compensation site with each method (A, B in Fig. 3), and then, using the estimations for the scenarios of the compensation site, we calculate the ecological state after the scenarios (D, E, F in Fig. 3). Tables 3 and 4 represent the results: the ecological state in each case as well as the amount of gain in each scenario as well as the resulting trade ratios following Eq. (6).

**Table 3:** Ecological state at the development and compensation sites before and after compensation

		The multiplicative method	The additive method	The matrix method
Development site	Current state	0.61	0.66	0.44
Compensation site	Current state	0.15	0.32	0.22
	Conservation	0.21	0.36	0.22
	Dead wood creation	0.43	0.55	0.44
	Burning	0.62	0.74	0.44

**Table 4:** The trade ratios with different calculation methods and different compensation scenarios

	The multiplicative method		The additive method		The matrix method	
	Gain	Trade ratio	Gain	Trade ratio	Gain	Trade ratio
Conservation	0.06	9.7	0.04	16.5	0	–
Dead wood creation	0.28	2.1	0.24	2.8	0.22	2.0
Burning	0.48	1.3	0.42	1.6	0.22	2.0

From Table 3 we can see that overall, the additive approach gives higher values for the ecological state than the multiplicative one. A similar finding was shown in “General Properties of the Multiplicative and Additive Methods” where the general properties of the methods were examined. Table 4 shows the trade ratios. The most notable difference between these methods is for the conservation scenario. The amount of gain attained is quite similar with both methods but as there is a clear difference in the state of the development site, i.e., the amount of loss (Table 3), and the amount of gain is very low, this leads to higher trade ratio with the additive method. Overall, the additive method results in higher trade ratios but in the other scenarios, the difference is more modest.

The result of the matrix method is derived from the condition score and distinctiveness score. As the scale for the condition score is quite coarse (0–3), it is not able to capture the difference between the baseline and conserving the site, or the scenarios of dead wood creation and prescribed burning. With conservation, there is no measurable gain with this calculation method. The restoration scenarios get the same score and thus result in the same trade ratio whereas, in the other two methods, there is a clear difference between the scenarios, and consequently the trade ratios are much higher in the case of dead wood creation.

### Offset Requirements with Adjusted Trade Ratios

Now we examine how adjusting the trade ratios with multipliers impacts the results. The use of multipliers varies depending on the offset mechanism. Therefore, we apply multipliers just as they are used in their original context in Finland, the state of Victoria in Australia, and the UK. Legislation or guidelines on offsetting are not yet established in Finland, but we follow Mustajärvi et al. (2019), who suggest addressing temporal issues with discounting in the ELITE index. Practices for considering other sources of uncertainty do not yet exist. In Victoria, where HH is used, different multipliers are applied for general habitat units and for general species units (DELWP 2017). The original UK matrix method employs a set of multipliers to account for time delays, the uncertainty of restoration, and the location of the compensation site (Crosher et al. 2019). The multipliers are described in more detail in Online Resource 2. Table 5 gives the time and risk-adjusted trade ratios from applying multipliers.

**Table 5:** Time and risk-adjusted trade ratios

	The multiplicative method (Finnish context)	The additive method (Victorian context)	The matrix method, time delay (UK context)	The matrix method, all multipliers (UK context)
Conservation	27.2	24.8	–	–
Dead wood creation	6.0	4.1	5.8	8.7
Burning	3.6	2.3	5.8	8.7

As the practices for using multipliers vary, comparing the results is not straightforward. However, trade ratios dealing with a time delay can be compared between the multiplicative method (discounts gain to net present value) and the matrix method (reduces the amount of gain by 0.343). Naturally, the trade ratios of both methods increase and exceed the ones of the additive method without multipliers. The order of the two methods stays the same because the ELITE index employs the same discount rate that was used to derive the time delay multiplier in the UK matrix method. With dead wood creation, the multiplicative method leads to a higher trade ratio (Table 4) and it is higher with the multipliers or discounting, too (Table 5). With burning, the matrix method gives a higher trade ratio (Table 4) and the same applies in Table 5. If all multipliers are included in the calculation of the matrix method, it leads to higher trade ratios in both restoration scenarios. The additive method accounts for both time delay and uncertainties of offset management in one multiplier (1.5). The order of trade ratios with the multiplicative and additive approach changes and the multiplicative approach leads to higher ratios. The same applies to the order of the additive and matrix methods. Thus, discounting the gains leads to a higher increase in the trade ratio than using a multiplier of 1.5 in the additive approach.

### Substitutability

In the literature, the question of substitutability is seen as a challenge in designing a calculation method (Gibbons et al. 2018; McCarthy et al. 2004). Substitutability means that the complete removal of one component can be offset perfectly by an increase in the abundance of another (Gibbons et al. 2018). This issue in the additive and multiplicative methods is examined by either elevating or degrading a single component and keeping other components constant. In order to take into account the role of weights, the results are calculated for a component with a low weight (decaying wood, stage 1) and a component with a high weight (broad-leaved trees). The values for the components as well as the results can be found in Online Resource 1.

The results suggest that there is a difference in how the multiplicative and additive approaches deal with substitutability. The multiplicative approach does not allow as much substitutability as the additive one, and the degree of substitution depends on the weights given for each component. When one component is degraded, the impact on the overall state is higher when calculated with the multiplicative method. When a component degrades to zero, with low weight, the overall state decreases 24% with the multiplicative method and 16% with the additive method. If a component with a high weight degrades, the impact is −40% with the multiplicative and −26% with the additive method. Thus, the weight of the component has a clear impact on substitutability—higher substitution is allowed if the weight is low. In contrast, if one component is restored to the reference state, the increase in overall state is more modest with the multiplicative method (9 and 16%) than with the additive method (16 and 26%).

### Costs for Developers

We next examine the costs of offsetting for the developer. The cost with each calculation method is calculated as costs per hectare × the trade ratio. The costs per hectare for conservation and the restoration measures were represented in “Hypothetical Offsetting Case and Scenarios” and trade ratios determining the size of the compensation site can be found in Table 4. Recall that we assume the area of the development site to be 1 ha. The costs of the restoration scenarios also include the cost of conservation. Table 6 represents the results.

**Table 6:** Costs of offsetting derived using trade ratios from Table 4

	Costs per ha, €	The multiplicative method	The additive method	The matrix method
Conservation	3000	28,590	48,730	–
Dead wood creation	3500	7380	9500	6890
Burning	4500	5690	6900	8890

The costs of offsetting vary significantly between the scenarios. Table 4 showed that the ecological gain of conservation is very low compared to restoration when measured with the multiplicative and additive methods. The high trade ratio of conservation leads to very high costs even though the cost per hectare is lower than with active restoration. This difference in costs is clearly demonstrated with the multiplicative and additive methods. They favor prescribed burnings and give very high costs to conservation. Even though burnings are costly to carry out, they are effective in restoring the components included in the methods, which is shown in the costs. The situation is different from the matrix method. It gives the same trade ratios as dead wood creation and burning. Unlike the other methods, trade ratios derived using the matrix method leads to favoring dead wood creation. From conservation, there was no calculable gain.

### Sensitivity analysis

We perform sensitivity analysis by considering the uncertainty of conservation and restoration outcomes as well as the determination of the baseline. Furthermore, we examine the sensitivity of the results to the weights of dead wood components. Each issue is examined independently and thus, the results can be compared to Tables 3 and 4 in “Offset Requirements in Different Scenarios”. Please see Online Resource 3 for a more detailed description of the methods. Detailed calculations can be found in Online Resource 1.

First, we examine the implications of using a dynamic baseline by replacing the fixed baseline of the main analysis. The baseline can be either increasing if the silvicultural management at the site is less intensive, (e.g., continuous cover forestry) or decreasing if the site is in intensive commercial use. The sensitivity analysis is performed by increasing or decreasing each component by 40% from the current state of the compensation site. The trade ratios change only when calculated with the multiplicative method: with conservation to 6.8 when the baseline is decreasing and to 17.2 when the baseline is increasing, with dead wood creation to 2.0 and 2.4 and with burning 1.2 and 1.4 (see detailed results in Online Resource 3).

Second, the dead wood components have a significant role in the calculations, both because they have the highest weight in total relative to other components and because the restoration measures impact mainly the amount of dead wood. In the sensitivity analysis the weights of dead wood are set to equal the weights of other components, i.e., the impact of having no dead wood is −0.4 to the overall condition. When the weights are changed, the current states of the development and compensation sites as well as the states in the different scenarios change. Lowering the dead wood weights and consequently diminishing the role of dead wood in the calculation decreases the amount of gain produced with restoration and conservation but does not change the results related to cost-effectiveness or the order of the methods: burning is still the most cost-effective scenario when the calculations are done with the multiplicative and additive method whereas creating dead wood is the most cost-effective option with the matrix method. The additive method provides the highest trade ratios apart from prescribed burning, where the matrix method leads to the highest one. See Online 3 for the detailed results.

Finally, in Table 7, we consider the uncertain outcomes of the three offset scenarios. The outcome of conservation is uncertain due to e.g., stochastic weather conditions and disturbances, such as insect outbreaks. When dead wood is artificially produced, its quality in comparison to naturally born dead wood may vary and similarly, the outcome of prescribed fire is dependent on the intensity of the fire, among other things. As literature does not provide exact figures to determine the scale of uncertainty values for the components, the sensitivity analysis is performed by increasing (upper bound) or decreasing (lower bound) each component from Table 2 by 40% and then calculating the ecological state.

**Table 7:** Sensitivity analysis for conservation and restoration uncertainty

	The multiplicative method		The additive method		The matrix method	
	Lower bound	Upper bound	Lower bound	Upper bound	Lower bound	Upper bound
Conservation	66.6	5.1	–	5.5	–	–
Dead wood creation	4.7	1.8	5.5	1.9	–	2.0
Burning	2.7	1.1	5.5	1.3	2.0	1.0

Table 7 represents the results. Considering the lower bound increases all trade ratios and has the most significant impact in the conservation scenario as the gain produced with only conserving the site is low in the main analysis as well. When the upper bound is examined, the trade ratios naturally decrease, but changes are more moderate. However, trade ratios in the conservation scenario drop notably, especially with the additive approach.

## Discussion

We compared different approaches to build a biodiversity offset calculation method: combining biodiversity components with a multiplicative or an additive function or using a simpler matrix method. We found that the outcomes of the calculation methods differ in many aspects. The results are in line with Bull et al. (2014a) who found significant differences between HH and the method developed in the UK and that their modified version of HH came closest to achieving NNL of biodiversity in their case study. Also, we find differences in cost-effectiveness when producing the required offset either with passive conservation or with active and costly restoration measures, an issue that has not been widely examined in the context of biodiversity offsetting.

Our results support the view that there are some downsides of using a simple approach like the matrix method even though it may be more straightforward and quicker to use than more complex ones (Treweek et al. 2010). However, it should be noted that the simplicity and labor intensity of the matrix method heavily depends on the metric which determines the condition score. The matrix approach is not capable of considering small changes in the ecological state due to the crude scale for the condition score (0–3). The choice of the scoring is subjective as there is no scientific support for a certain scale. Yet the scoring has a significant impact on the results. Using a crude method such as this creates a risk that important biodiversity features are lost in the offset trade. Better accuracy, as well as repeatability and transparency, are strengths of the other two approaches as there is less subjectivity in the calculation (Koh et al. 2014; McCarthy et al. 2004). However, as the multiplicative and additive methods use several habitat components, they are more intensive in terms of the data required to assess the ecosystem. We have used the multiplicative method as a basis for the condition score in the matrix method, but the score could be measured with a simpler method and thus, a lesser amount of data would suffice.

We used scoring intervals when building the additive approach, following HH. Our results suggest that using continuous data as in the multiplicative approach or categorizing data with scoring intervals as in the additive approach may highlight the difference between these two methods. The additive function leads to higher biodiversity values and categorizing the field assessment data into scores may further increase the difference. For instance, if the amount of decaying wood in stage 2 would increase by 1 m^3^, the total value would increase by ~2% with the multiplicative method, but with the additive one, it would depend on the value and the number of categories. In our analysis, if the state increased from 3 to 4 m^3^, the overall state would not change. If the increase was from 7 to 8 m^3^, the overall state would increase ~13%. Thus, the use of scoring intervals in the additive method has a risk of reducing sensitivity to real differences. This issue is demonstrated in the sensitivity analysis for the baseline, where the changes in the values of the components are moderate and thus, the additive and matrix methods show no change in the calculation results, whereas the multiplicative method recognizes these moderate changes. HH uses visual assessments, which may justify the use of intervals to decrease the impact of subjectivity and reduce inaccuracies. Using continuous components that can be objectively measured may be more accurate than visual assessments (Gorrod and Keith 2009; McCarthy et al. 2004) and the need to specify subjective thresholds are removed.

The calculation methods have differences in dealing with the substitutability of individual components. The matrix method assumes that all biodiversity values are commensurable (Treweek et al. 2010), although, this is dependent on the metric the condition score is calculated with. The additive function allows substitutability between different components (McCarthy et al. 2004), whereas, in the ELITE index, the use of multiplicative function aims to reduce substitutability. With the additive approach, the landowner would need to restore one component in order to attain a certain amount of gain, while using the multiplicative approach, the landowner would need to restore other components as well to attain the same amount of gain. Thus, a calculation method that allows components to be substituted may lead to a situation where components that are easy to restore are preferred at the expense of those that are more difficult to restore (Gibbons et al. 2018). Still, the multiplicative approach allows some degree of substitutability. The only way to eliminate the risks of substitutability completely is to use separate metrics for each biodiversity component (Maseyk et al. 2016). Finding an equivalent offset to match many different currencies would be challenging. Aggregating different components into one currency is needed to ensure feasibility, but it is important to find a balance so that the impacts we aim to compensate are not aggregated in such a way that unintended substitution can occur.

There are multiple aspects of calculating biodiversity offsets that we did not consider or examined only briefly, including uncertainty, temporal and spatial issues, and potential leakage of impacts (Moilanen and Kotiaho 2018). In addition, our analysis did not consider the incentives of landowners. The choice of a calculation method has an impact on what kind of gains the landowner would provide as it is beneficial to restore only the features of biodiversity that are measured. The choice of components impacts the incentives greatly. Also, the weights of the components matter as enhancing some components increases the condition more than others. The costs of restoration play an important role: landowners restore those components that are most cost-effective to restore. This is shown in our results as the volumes of dead wood in different decay stages have in total the highest weight and thus, restoration measures that effectively increase dead wood volume are probably the most cost-effective option to produce offsets. With the matrix method, the landowner is not incentivized to guarantee that the ecosystem reaches its natural state or as good a state as possible as the highest amount of gain is attained when the condition score of the site reaches category “high”. In our analysis, this corresponds to index value 0.67 with the multiplicative method. However, gains can also be generated by elevating the distinctiveness category, where possible.

The amount of dead wood has a dominating role in the calculations, both because the components of dead wood in total have the highest weight relative to other components and because the restoration measures have the strongest impact on the amount of dead wood. In natural boreal forests, the amount and type of dead wood are one of the most important structural components (Stokland et al. 2004): on average 25% of above-ground wood biomass is dead wood in natural forests, and it is a key factor in species diversity (Siitonen 2001). Thus, dead wood is often considered as an indicator of forest naturalness and biodiversity in the boreal zone (Halme et al. 2019; Kunttu et al. 2015; Similä et al. 2006). In addition to dead wood volume, the quality of dead wood should be considered (Halme et al. 2019; Stokland et al. 2004), which we have done by using decay stages. However, the dominating role of dead wood in the calculations and the rather limited number of components decrease the generalizability of the results outside the boreal zone concerning the cost-effectiveness of different measures of producing the offset. There are many proxies for forest biodiversity that could be incorporated into the methods (e.g., the successional stage, microclimate, seedling recruitment, herbivory) (Similä and Junninen 2012; Tikkanen et al. 2006), which might change the performance of different scenarios in producing offset gains. Furthermore, the results should be generalized with caution also because we used only two sites in our analysis.

How the calculation method in a biodiversity offsetting scheme is developed has a significant impact on the resulting offset requirements. When designing a calculation method, trade-offs between simplicity and ecological robustness as well as a more detailed method and efficiency must be balanced. When making these decisions, it is important to acknowledge the implications of these trade-offs. As there is no comprehensive calculation method (Bezombes et al. 2017), the strengths and weaknesses of the chosen method must be openly communicated.

## Supplementary information

Online Resource 1

Supplementary Material 1

Supplementary Material 2

## Data Availability

Data presented in the main manuscript as well as in the supporting materials (Online Resource 1).

## Footnotes

1 The R code for producing the outputs and detailed methodology can be accessed from https://github.com/PKullberg/compensation_method_comparison
