# Supplementary material for: Precision, Applicability, and Economic Implications: A Comparison of Alternative Biodiversity Offset Indexes
Source: Environ Manage. 2021 Jun 7;68(2):170–83. doi: 10.1007/s00267-021-01488-5 (PMC8241746; doi:10.1007/s00267-021-01488-5)
Supplement: Supplementary file 2 — Supplementary Material 1 [file 267_2021_1488_MOESM2_ESM.pdf]

Online Resource 2: Multipliers

Precision, applicability and economic implications: A comparison of alternative biodiversity offset indexes

Environmental Management

Johanna Kangas, Peter Kullberg, Minna Pekkonen, Janne S. Kotiaho, Markku Ollikainen

Corresponding author: Johanna Kangas, johanna.a.kangas@helsinki.fi, Department of Economics and Management, University of Helsinki

Tables 2.1-2.3 show the multipliers employed in the UK method. They use multipliers to reduce the amount of gain generated with conservation and restoration (Eq. (2.1)). The multiplier for risks related to the success of restoration is applied to the Finnish ecosystems based on Raunio et al. (2019). The multipliers employed to our analysis are 0.343 for time delay, 0.67 for restoration uncertainty and 1 for location.

$$\sigma = \frac{L}{\mathrm{G} m_1 m_2 m_3} \tag{2.1}$$

where m_1, m_2, m_3 are the multipliers for temporal risk, restoration risk and spatial risk.

Table 2.1 Multipliers for temporal risk. Adapted from Crosher et al. (2019)

Years to target condition	*Multiplier*
0	1
5	0.837
10	0.700
15	0.586
20	0.490
25	0.410
30	0.343

Table 2.2 Multipliers for restoration risk. Adapted from Crosher et al. (2019)

Difficulty of restoration	*Multiplier*
Low	1
Medium	0.67
High	0.33
Very high	0.1

Table 2.3 Multipliers for spatial risk. Adapted from Crosher et al. (2019)

Location	*Multiplier*
Offset in a geographical area identified in the offsetting strategy	1
Offset outside, but neighboring an area identified in the offsetting strategy	0.75
Offset outside and beyond neighboring an area identified in the offsetting strategy	0.5

In Victoria, Australia with Habitat Hectares, one multiplier is included when calculating the required amount of offset to account for both restoration uncertainty and time delay (Eq. (2.2)). The multiplier is designed to balance the risk that some of the predicted gains are not realised at the compensation site and also that the immediate loss is offset with gains that are expected to be realised in the future (DELWP 2017). A multiplier of 1.5 is applied to all general offsets (the degradation does not have a significant impact on any habitat for rare or threatened species) and a multiplier of 2 is applied to all species offsets (DELWP 2017). Thus, we employ a multiplier of 1.5.

$$\sigma = k\frac{L}{\mathrm{G}(1+r)^{-t}} \tag{2.2}$$

where k is the multiplier.

ELITE index uses discounting to measure the net present value of the offset gain when there is time delay (Mustajärvi et al. 2019). The discount factor increases the trading ratio whenever there is temporal loss before gains mature. Trading ratio is then calculated as follows:

$$\sigma = \frac{L}{\mathrm{G}(1+r)^{-t}}, \tag{2.3}$$

where r is the discount rate (3.5%, following Crosher et al. (2019) and t is the amount of time delay in years (30).
