# Supplementary material for: Precision, Applicability, and Economic Implications: A Comparison of Alternative Biodiversity Offset Indexes
Source: Environ Manage. 2021 Jun 7;68(2):170–83. doi: 10.1007/s00267-021-01488-5 (PMC8241746; doi:10.1007/s00267-021-01488-5)
Supplement: Supplementary file 3 — Supplementary Material 2 [file 267_2021_1488_MOESM3_ESM.pdf]

Online Resource 3: Sensitivity analysis

Precision, applicability and economic implications: A comparison of alternative biodiversity offset indexes
Environmental Management

Johanna Kangas, Peter Kullberg, Minna Pekkonen, Janne S. Kotiaho, Markku Ollikainen
Corresponding author: Johanna Kangas, johanna.a.kangas@helsinki.fi, Department of Economics and Management, University of Helsinki

Dynamic baseline instead of a fixed one

In the main analysis, we assumed the baseline to be fixed and thus, the components to remain fixed in the current state. The implications of using a dynamic baseline are examined by increasing or decreasing each component by 40 % from the current state of the compensation site. The number of large trees is kept constant as in the main analysis. The results with an increasing baseline are represented in Table 3.1 and with decreasing baseline are represented in Table 3.2.

Table 3.1 Sensitivity analysis for baseline uncertainty, increasing baseline

	**The multiplicative method**		**The additive method**		**The matrix method**	
	Gain	*Trade ratio*	*Gain*	*Trade ratio*	*Gain*	*Trade ratio*
Conservation	0.04	17.2	0.04	16.5	0	-
Dead wood creation	0.26	2.4	0.24	2.8	0.22	2.0
Burning	0.42	1.4	0.42	1.6	0.22	2.0

Table 3.2 Sensitivity analysis for baseline uncertainty, decreasing baseline

	**The multiplicative method**		**The additive method**		**The matrix method**	
	Gain	*Trade ratio*	*Gain*	*Trade ratio*	*Gain*	*Trade ratio*
Conservation	0.09	6.8	0.04	16.5	0	-
Dead wood creation	0.31	2.0	0.24	2.8	0.22	2.0
Burning	0.5	1.2	0.42	1.6	0.22	2.0

Weights for dead wood

Sensitivity analysis for the weights of different stages of dead wood was performed by decreasing the weights so that the impact of having no dead wood at all is -40% to the overall state, which is equal to the impact of other components. Thus, the impact of one decay stage is -14%. In the main analysis, the impact is -60% for dead wood in total and -24% for one decay stage. This has an impact to the values of current states of the development site and the compensation site (Table 3.3) as well as values in different scenarios (Table 3.4).

Table 3.3 Sensitivity analysis for dead wood weights, condition in current state

	**The multiplicative method**	**The additive method**	**The matrix method**
Development site	0.74	0.72	0.67
Compensation site	0.21	0.33	0.22

Table 3.4 Sensitivity analysis for dead wood weights, condition after scenarios

	**The multiplicative method**			**The additive method**			**The matrix method**		
	State	*Gain*	*Trade ratio*	*State*	*Gain*	*Trade ratio*	*State*	*Gain*	*Trade ratio*
Conservation	0.27	0.05	14.1	0.36	0.03	24.9	0.22	0	-
Dead wood creation	0.45	0.24	3.1	0.50	0.17	4.2	0.44	0.22	3.0
Burning	0.61	0.39	1.9	0.67	0.34	2.1	0.44	0.22	3.0

Uncertainty of the impacts of conservation and restoration

As literature does not provide figures to determine the upper and lower bounds for the components in different management scenarios, the sensitivity analysis is performed by increasing (upper bound) or decreasing (lower bound) each component by 40 % and calculating the ecological state after the decrease or increase of the components. The number of large trees is kept constant as in the main analysis. The results are represented in Table 3.5 and 3.6. The detailed calculations can be found in Online Resource 1.

Table 3.5 Sensitivity analysis for conservation and restoration uncertainty, lower bound

	**The multiplicative method**			**The additive method**			**The matrix method**		
	State	*Gain*	*Trade ratio*	*State*	*Gain*	*Trade ratio*	*State*	*Gain*	*Trade ratio*
Conservation	0.16	0.01	66.6	0.32	0	-	0.22	0	-
Dead wood creation	0.28	0.13	4.7	0.43	0.12	5.5	0.22	0	-
Burning	0.37	0.22	2.7	0.43	0.12	5.5	0.44	0.22	2.0

Table 3.6 Sensitivity analysis for restoration uncertainty, upper bound

	**The multiplicative method**			**The additive method**			**The matrix method**		
	State	*Gain*	*Trade ratio*	*State*	*Gain*	*Trade ratio*	*State*	*Gain*	*Trade ratio*
Conservation	0.27	0.12	5.1	0.43	0.12	5.5	0.22	0	-
Dead wood creation	0.48	0.33	1.8	0.66	0.34	1.9	0.44	0.22	2.0
Burning	0.69	0.54	1.1	0.80	0.49	1.3	0.67	0.44	1.0
